# 141 Implementation of exercise program after multidisciplinary treatment of chronic widespread musculoskeletal pain in the primary healthcare – study protocol

**DOI:** 10.1093/eurpub/ckae114.173

**Published:** 2024-09-26

**Authors:** Suzana Pustivšek, Maja Dakskobler, Andraž Furlan

**Affiliations:** National Institute Of Public Health Of Slovenia, Ljubljana, Slovenia; National Institute Of Public Health Of Slovenia, Ljubljana, Slovenia; Faculty of Sport, University of Ljubljana, Ljubljana, Slovenia

## Abstract

**Purpose:**

Chronic musculoskeletal pain (CMS) is affecting approximately one third of the adult population. There is a lack of evidence in the literature for multicomponent exercise programmes for patients with CMS pain and adherence to these programmes is rarely reported. The main research question is: Does the supervised, individualised, patient-centered exercise programme implemented following a multidisciplinary programme for the treatment of CMS pain have an additional impact on patients’ perceptions of their own health and well-being and adherence to physical activity?

**Methods:**

The study is a cohort study; the estimated sample size is 140 subjects. Participants will receive 16 supervised sessions. The first and last session will be individual, the other 14 will be group sessions with 8 participants led by a kinesiologist. Each session is divided in two parts: First includes warm-up and functional exercises, second includes breath-centered yoga practice with deep relaxation. Both, functional exercises and yoga practice will be graded through the sessions, with particular attention paid to the individualization of the participants. Outcome assessments will occur at baseline (1st session), end of intervention (16th session) and 8 weeks after the intervention. The main parameters will be patients’ perception of their own health and well-being (SF – 12 questionnaire), exercise adherence (ATEMPT questionnaire), physical fitness (senior fitness test battery) and pain level (numerical rating scale). Pre-specified analyses will evaluate the main effects of the treatment by comparing pre, post and follow-up results.

**Results:**

The study will start in April 2024, with first preliminary results expected by the end of the year.

**Conclusion:**

This research will use a novel approach to treating CMS pain, as a programme combining graded functional exercises and breath-centered yoga practice will be an extension of multidisciplinary CMS pain management. If the programme shows significant benefits for users, it can be used as a maintenance programme for an active lifestyle for people with CMS pain. In this way, the burden of CMS pain on the healthcare sector can be reduced.

**Fundings:**

The project “Upgrading treatment of musculoskeletal pain at the primary level” is being implemented within the framework of the Recovery and Resilience Plan.

